# Subtyping Early Parkinson’s Disease by Mapping Cognitive Profiles to Brain Atrophy with Visual MRI Ratings

**DOI:** 10.3390/brainsci15070751

**Published:** 2025-07-15

**Authors:** Tania Álvarez-Avellón, Carmen Solares, Juan Álvarez-Carriles, Manuel Menéndez-González

**Affiliations:** 1 Departamento de Psicología, Universidad de Oviedo, 33009 Oviedo, Spain; 2 Instituto de Investigación Sanitaria del Principado de Asturias, 33011 Oviedo, Spain; 3 Servicio de Psiquiatría de Enlace, Hospital Universitario Central de Asturias, 33009 Oviedo, Spain; 4 Departamento de Medicina, Universidad de Oviedo, 33006 Oviedo, Spain; 5 Servicio de Neurología, Hospital Universitario Central de Asturias, 33011 Oviedo, Spain

**Keywords:** Parkinson’s disease, cognitive decline, neuroanatomy, structural MRI, visual atrophy scales

## Abstract

Background: Cognitive heterogeneity in Parkinson’s disease (PD) remains a diagnostic and prognostic challenge, particularly in early stages. In this cross-sectional study, we aimed to identify clinically relevant cognitive subtypes in early PD by integrating neuropsychological profiles with regional brain atrophy assessed via visual MRI scales. Methods: Eighty-one de novo PD patients (≤36 months from diagnosis) and twenty healthy controls underwent 3T MRI with visual atrophy ratings and completed an extensive neuropsychological battery. Results: Using a mixed a priori–a posteriori approach, we defined eight anatomocognitive subtypes reflecting distinct patterns of regional vulnerability: frontosubcortical, posterior cortical, left/right hippocampal, global, and preserved cognition. Specific MRI markers correlated with cognitive deficits in executive, visuospatial, memory, and language domains. Cluster analyses supported subtype validity (AUC range: 0.68–0.95). Conclusions: These results support a practical classification model linking cognitive performance to brain structural changes in early PD. This scalable approach may improve early patient stratification and guide personalized management strategies. Longitudinal studies are needed to assess progression patterns and therapeutic implications.

## 1. Introduction

Parkinson’s disease (PD) is increasingly understood as a multidimensional neurodegenerative disorder, in which cognitive impairment represents a core, disabling domain. Following James Parkinson’s 1817 description, initially centered on motor symptoms, research has underscored the prevalence and prognostic significance of non-motor manifestations, particularly cognitive decline [1,2,3]. Estimates suggest that over 30% of patients develop mild cognitive impairment (PD-MCI) within the first three years, with up to 70% progressing to dementia (PDD) during the disease course [4,5,6].

Cognitive dysfunction in PD is highly heterogeneous, typically involving executive deficits, visuospatial impairments, episodic memory loss, and language alterations [7,8]. This variability reflects diverse neuropathological substrates, including dopaminergic and non-dopaminergic degeneration, α-synucleinopathy, and frequent co-pathology with Alzheimer’s disease [9,10]. Pinpointing cognitive subtypes is essential for a deeper understanding of their underlying pathophysiology, for predicting how the disease will evolve, and for crafting personalized treatment strategies. Different classifications of cognitive subtypes have been described that differentiate between PD-MCI and more advanced stages, including PDD, with a strong emphasis on their neuroanatomical and neuroimaging underpinnings.

In the early phases of PD, PD-MCI presents as cognitive deficits that do not yet meet the criteria for dementia. Classifications at this stage often hinge on the specific cognitive domains affected, differentiating between single-domain (e.g., visuospatial) and multiple-domain impairments [11]. Beyond broad categories, some research has identified more refined neuropsychological subtypes within PD-MCI, notably those with predominantly fronto-executive or visuospatial deficits. Neuroimaging techniques, such as functional magnetic resonance imaging (fMRI) or 18F-FDG positron emission tomography (PET), frequently reveal corresponding patterns of hypometabolism or dysfunction within specific brain networks, including the default mode network and frontoparietal network, which are closely linked to these neuropsychological profiles [12,13].

As PD progresses, cognitive impairment can escalate to PDD. Classifications in these later stages become more intricate, often aiming to predict the trajectory of cognitive decline. Specific dementia profiles emerge, such as dementia with a predominance of executive/frontosubcortical dysfunction, characterized by difficulties in planning and attention, often accompanied by changes in dopaminergic pathways and frontal and subcortical regions. Another profile is dementia with a predominance of visuospatial dysfunction, commonly associated with visual hallucinations. While less typical in pure PD, a dementia profile featuring predominant memory deficits (Alzheimer-like type) can occur, particularly when there’s coexisting amyloid or tau pathology. Neuroimaging plays a crucial role here: 11C-PIB PET for amyloid and 18F-AV-1451 PET for tau can help distinguish subtypes with additional pathologies, and distinct patterns of regional cortical atrophy on structural MRI are often indicative of different clinical presentations [14,15]. Furthermore, data-driven approaches using machine learning have identified specific patterns of brain atrophy (from structural MRI) or hypometabolism (from 18F-FDG PET) that correlate with diverse cognitive profiles and progression rates. These studies have highlighted atrophy patterns affecting areas such as the posterior cortex or frontotemporal regions, which are associated with varying cognitive outcomes and prognoses [16,17]. Recent investigations have also sought to differentiate between rapid and slow cognitive decline subtypes in PD, often by analyzing the rate of change in neuropsychological scores over time. These distinct trajectories are frequently correlated with specific neuroimaging biomarkers, such as reduced white matter integrity as assessed by diffusion tensor imaging (DTI) and increased hippocampal atrophy on structural MRI [18]. A systematic review evaluated in 2022 data-driven studies on cognitive subtypes in PD: a total of 22 articles were identified, revealing subtype structures that ranged from a spectrum of severity to domain-specific patterns of impairment, including amnestic/nonamnestic, memory/executive, and frontal/posterior dichotomies. Preliminary longitudinal data suggested differential cognitive trajectories across subtypes, while neuroimaging studies highlighted distinct patterns of brain alterations. The findings underscore the potential clinical relevance of cognitive subtyping in PD and call for further research integrating neuroimaging to elucidate underlying neural mechanisms. Despite the lack of consensus, the literature shows increasing consistency and utility in defining cognitive subtypes [19]. 

Despite significant advances, there remains a lack of practical, reproducible classification systems linking cognitive phenotypes to structural brain changes, particularly in early-stage PD. While quantitative MRI volumetry has contributed valuable insights, it is often resource-intensive and not widely available in clinical settings. In contrast, visual atrophy scales offer a cost-effective and clinically accessible alternative, with demonstrated utility in Alzheimer’s disease [20,21] and emerging relevance in PD.

As can be seen, several efforts have proposed data-driven cognitive subtyping approaches in PD; however, many lack a clear anatomical grounding, limiting their interpretability and applicability in clinical practice. In the absence of a recognizable localizational framework, these models often provide limited guidance for understanding the neurobiological basis of cognitive impairment. A classification anchored in classic neuroanatomical–functional correspondences could serve as a practical “cognitive map,” bridging research findings with everyday diagnostic reasoning and supporting more precise patient stratification.

## 2. Materials and Methods

### 2.1. Participants

Eighty-one patients diagnosed with idiopathic Parkinson’s disease (PD), according to the UK Parkinson’s Disease Society Brain Bank Criteria, were consecutively recruited from the Movement Disorders Unit at the University Hospital of Asturias (HUCA) between March 2015 and June 2019. All patients were assessed within 36 months of diagnosis, and they were receiving their usual treatment at the time of enrollment, as recommended by their neurologists. A control group of twenty healthy individuals was selected among those referred for benign neurological complaints, such as tension-type headache or peripheral paresthesia, who presented normal clinical evaluations and brain MRI findings. Inclusion criteria for the PD group comprised fulfillment of the diagnostic criteria for idiopathic PD, a disease duration of 36 months or less, and availability of a 3-Tesla MRI within 12 months of cognitive testing. Exclusion criteria for both groups included a prior history of dementia, stroke, traumatic brain injury, epilepsy, or multiple sclerosis; presence of major psychiatric disorders such as schizophrenia or bipolar disorder; contraindications to MRI; or a Mini-Mental State Examination (MMSE) score below 24. All participants provided written informed consent. The study was approved by the HUCA Ethics Committee (approval number 73/15, date of approval June 2015).

### 2.2. Neuropsychological Assessment

Cognitive testing was conducted in two sessions of approximately 90 min each, scheduled 5 to 10 days apart. Participants were assessed in the “ON” medication state. Global cognition was evaluated using the Montreal Cognitive Assessment (MoCA) and the Parkinson’s Disease Cognitive Rating Scale (PD-CRS). Specific cognitive domains were explored through a comprehensive neuropsychological battery. Executive function was assessed using the Tower of Hanoi for planning and problem-solving, the three conditions of the Stroop test (word, color, and interference), action fluency and naming tasks, as well as phonemic, semantic, and alternating verbal fluency. Additional executive subtests included abstraction and verbal reasoning items from the MoCA. Attention and working memory were examined using forward and backward digit span, serial 7 s subtraction, the Symbol Digit Modalities Test (SDMT), and the Trail Making Test parts A and B. Visuospatial abilities were evaluated using the Judgment of Line Orientation (JLO), the Rey–Osterrieth Complex Figure Copy, and the Clock Drawing Task from the MoCA. Episodic memory was assessed using the Hopkins Verbal Learning Test–Revised (HVLT-R), including immediate recall, delayed recall, and recognition. Raw test scores were adjusted for age and education using available normative data. Standardized z-scores were calculated and used in subsequent multivariate analyses.

### 2.3. MRI Acquisition and Visual Rating

All MRI scans were acquired using the same 3-Tesla Philips scanner with a T1-weighted MPRAGE protocol and 1 mm isotropic voxels. A single neurologist trained in visual MRI rating, blinded to all clinical and cognitive information, performed regional atrophy assessments using validated visual scales. The medial temporal lobe was rated using the Scheltens Medial Temporal Atrophy (MTA) scale (range 0–4), posterior cortical atrophy was evaluated using the Koedam Posterior Atrophy (PA) scale (range 0–3), and frontal lobe atrophy was assessed with the Global Cortical Atrophy–Frontal (GCA-F) subscale (range 0–3). In addition, three bilateral visual scales developed by Harper et al. [20,21] were applied, namely orbitofrontal, anterior cingulate, and frontoinsular cortex atrophy, each scored on a 4-point scale. Ratings were performed independently for each hemisphere.

### 2.4. Statistical Analysis

All analyses were conducted in R 4.3.1 and Python 3.9. Results were considered significant at *p* < 0.05, except in analyses corrected for multiple comparisons (Bonferroni and FDR). The sample was described using means, standard deviations, and frequencies. Cases, controls, and PD-CRS subgroups were compared using *t*-tests, ANOVA, and MANOVA applied to neuropsychological measures, visual atrophy scales, and cortical volumes. Relationships between cognitive, demographic, and neuroimaging variables were assessed with correlation analyses, while multiple linear and logistic regression models identified predictors of cognitive impairment and their neuroimaging correlates.

Normality of variables was assessed using Shapiro–Wilk tests: patient age was not normally distributed (unlike controls), MoCA scores deviated from normality in the control group, and the cortical subtotal of the PD-CRS was also non-normal. Consequently, for two-group comparisons, the Mann–Whitney U test was used when normality was not met, and Student’s *t*-test with Welch’s correction was applied in parametric cases.

For multivariate models, categorical variables (e.g., education level) were one-hot encoded, and missing data (<1%) were imputed using k-nearest neighbor imputation (*k* = 5) within cross-validation folds to prevent information leakage. Neuropsychological scores were z-score normalized within the training folds.

The LASSO regularization parameter (lambda) was optimized via 10-fold nested cross-validation using the glmnet package. Standardized beta coefficients were averaged across 100 bootstrap iterations; predictors were considered robust if non-zero coefficients appeared in ≥80% of iterations with |β| > 0.25. Residual normality and homoscedasticity in regression models were assessed with Shapiro–Wilk and Levene’s tests, respectively; parametric tests were applied where assumptions held, and sensitivity analyses confirmed robustness where deviations occurred.

For clustering, stability was assessed using random centroid initialization (k-means++), achieving high consistency across 100 iterations (adjusted Rand index = 0.87). Validation metrics included internal measures (silhouette score, Dunn index) and external checks for biological plausibility, such as alignment with known neurodegenerative patterns. Discriminative performance was evaluated via one-vs-rest ROC curves in leave-one-out cross-validation, and AUC values were interpreted using standard thresholds (acceptable: >0.7; good: >0.8).

## 3. Results

### 3.1. Demographic and Clinical Characteristics

The final sample included 81 individuals diagnosed with early-stage Parkinson’s disease (PD) and 20 cognitively healthy controls. The mean age in the PD group was 66.4 years (SD = 7.9), compared to 62.0 years (SD = 8.1) in the control group; normality was confirmed with the Shapiro–Wilk test, and this difference was not statistically significant (t = 1.62, *p* = 0.119). Sex distribution was balanced across groups, with the PD group consisting of 45 males and 36 females, and the control group comprising 8 males and 12 females (χ^2^ = 0.99, *p* = 0.3185). Right-handedness predominated in both cohorts, with no relevant asymmetry in laterality. No significant difference emerged in educational attainment between groups (χ^2^ = 2.6107, *p* = 0.2711). This variable was considered in subsequent analyses. Table 1 summarizes the main demographic and clinical features of both groups.

### 3.2. Global Cognitive Performance

Patients with PD exhibited significantly reduced performance on global cognitive screening measures relative to healthy controls. The mean MoCA score in the PD group was 22.1 (SD = 4.2), significantly lower than the 27.2 (SD = 1.7) observed in controls (*p* < 0.001). Similarly, PD patients scored significantly lower on the PD-CRS (mean = 82.7, SD = 15.8) compared to controls (mean = 117.2, SD = 7.2), with *p* < 0.001.

### 3.3. Neuropsychological Correlations

The Bonferroni-corrected Pearson correlations between the neuropsychological tests and the three composite scores of the PD-CRS are shown in Table 2. Among the most significant associations, SDMT showed the strongest associations with the sub-cortical (r = 0.856), cortical (r = 0.595), and total (r = 0.862) subscores, highlighting its relationship with psychomotor speed and global cognitive status. Phonemic fluency also showed strong correlations with the three dimensions (r = 0.789; 0.458; 0.781), as did action fluency (r = 0.750; 0.395; 0.737) and semantic fluency (r = 0.664; 0.376; 0.656). In the executive domain, the number of moves in the Tower of Hanoi was negatively associated with the subcortical (r = −0.433), cortical (r = −0.420), and total (r = −0.444) scores, while the resolution time yielded even higher negative coefficients (r = −0.658; −0.520; −0.663). The Judgment of Line Orientation test reached very high correlations with the three subscores (r = 0.754; 0.504; 0.756), reflecting its sensitivity to visuospa-tial abilities.

### 3.4. Age and Education Effects on Cognition

Regression analyses revealed that advancing age was significantly associated with poorer performance in tasks demanding executive control and processing speed. Specifically, older individuals performed worse on the Stroop interference task and the Tower of Hanoi, with standardized beta coefficients of −0.31 and −0.28, respectively (both *p* < 0.01). In contrast, years of formal education, treated as an ordinal variable in the regression models, correlated positively with phonemic fluency and digit span performance (β = 0.34 and 0.29, respectively; *p* < 0.01), underscoring a protective role of cognitive reserve.

### 3.5. Brain Atrophy and Cognitive Correlates

Atrophy-cognitive correlations are presented as complementary descriptive anal-yses and do not constitute an independent validation of the clustering framework: par-tial correlation analyses, adjusted for age and education, demonstrated significant as-sociations between performance on domain-specific neuropsychological tests and re-gional brain atrophy assessed by visual MRI rating scales. Significant associations be-tween regional atrophy and neuropsychological performance were observed bilateral-ly. Greater medial temporal atrophy (MTA), posterior atrophy (PA), and orbitofrontal cortical atrophy were significantly associated with lower global cognitive perfor-mance, as measured by the PD-CRS, with correlation coefficients of r = −0.42, −0.48, and −0.55, respectively (all *p* < 0.01). These findings reinforce the role of these regions in global cognitive functioning in PD.

In the right hemisphere, visual MRI ratings correlated with deficits in executive, visuospatial, and global cognition (Supplemental Table S1); analogous associations were found for the left hemisphere (Supplemental Table S2), spanning cortical and subcortical areas.

To assess group-level differences in cortical atrophy, a MANOVA comparing all visual ratings between PD patients and healthy controls showed significantly greater atrophy in the patient group (Supplemental Table S3). The most pronounced differences were found in the left orbitofrontal cortex, followed by the left medial and right anterior temporal cortices. Additional frontoinsular and anterior cingulate regions were also significantly affected, whereas posterior cortex changes, though present, were comparatively milder.

Stratifying patients into cognitively intact, MCI, and dementia subgroups revealed a stepwise atrophy gradient in eight out of twelve regions (Supplemental Table S4). The strongest effects emerged in the right anterior cingulate, right frontoinsular, and right medial temporal cortices. Other frontoinsular and temporal regions showed similar trends. In contrast, posterior cortical atrophy did not reach significance after correction, suggesting that progression toward dementia is primarily driven by fronto-cingulate and medial temporal degeneration.

Finally, one-vs-all Pearson correlations between visual atrophy ratings and PD-CRS composite scores demonstrated robust negative associations across both hemispheres (Supplemental Table S5A,B). The medial temporal, orbitofrontal, frontoinsular, and anterior cingulate regions showed the strongest correlations with total, frontosubcortical, and posterior PD-CRS scores, supporting the link between regional atrophy and cognitive dysfunction in early PD.

### 3.6. Subtype Classification Based on Cognitive–Anatomical Profiles and Discriminative Validity

The constrained clustering approach produced eight cognitive–anatomical subtypes characterized by distinct neuropsychological and regional atrophy profiles. Supplementary Table S6 shows the most informative tests and subtests for each anatomo-cognitive subtype. Details of cluster centroids and neuropsychological profiles are provided in Supplementary Tables S6 and S7 and Figures S1 and S2. The subtypes displayed anatomically plausible patterns, with left and right frontosubcortical clusters exhibiting predominant deficits in executive functions and corresponding frontoinsular atrophy, and hippocampal clusters showing selective memory impairment associated with medial temporal atrophy. Subtypes such as the left and right frontosubcortical groups exhibited higher median scores in orbitofrontal, frontoinsular, and anterior cingulate regions, consistent with executive dysfunction. In contrast, left and right hippocampal subtypes showed selective medial temporal atrophy and impaired memory performance. The global subtype displayed diffuse, multiregional atrophy involving frontal, temporal, and posterior cortices.

Silhouette analysis yielded a mean silhouette score of 0.52 (Supplementary Figure S2), indicating moderately well-defined clusters and supporting the internal consistency of the subtype structure. Most participants were tightly clustered around centroids, but seven individuals (8.6% of the PD cohort) exceeded 1 SD Mahalanobis distance from their assigned centroid and were classified as “atypical.” These individuals showed diffuse cognitive deficits and multiregional atrophy and were retained in group-level analyses. Sensitivity analyses using random centroid initialization (k-means++) yielded highly similar clustering solutions, with an adjusted Rand index of 0.87 across 100 iterations, supporting the robustness of the subtype structure.

**Figure 1:**
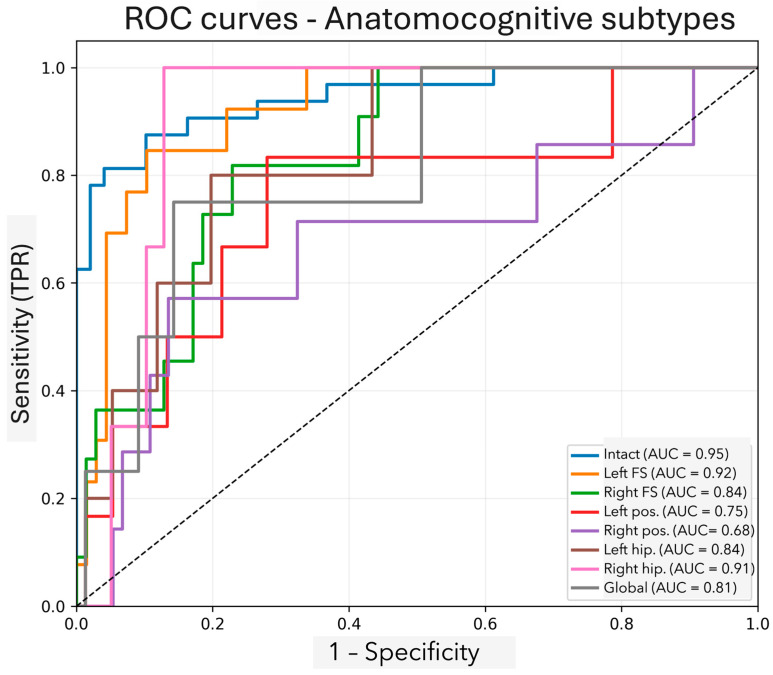
ROC curves of the cognitive subtypes. The curves illustrate the discriminative ability of the models trained for each anatomo-cognitive subtype. The areas under the curve obtained closely match those reported in the empirical analyses (0.68–0.95) and reflect an overall “acceptable-to-high” performance, with a clear separation from the chance diagonal. The highest trajectories correspond to the “cognitively intact” phenotype (AUC ≈ 0.95), whereas the most modest curve is seen for the right posterior subtype (AUC ≈ 0.68).

To assess the discriminative power of each subtype, we applied one-vs-rest receiver operating characteristic (ROC) analysis with leave-one-out cross-validation. The area under the curve (AUC) values ranged from 0.68 (left posterior subtype) to 0.95 (global subtype), reflecting moderate to high discriminative performance in post hoc analyses (Figure 1). The intact cognition group, used as a comparative reference, showed a lower AUC (0.65), consistent with its characterization as a normative baseline rather than a discriminative category.

These findings support the internal coherence and construct validity of the proposed classification model. The observed AUC range, particularly the strong discriminability of the global, right hippocampal, and frontoinsular subtypes, reinforces the potential clinical utility of this approach for early patient stratification. Nevertheless, future validation in independent and longitudinal datasets is necessary to establish prognostic stability and translational value.

The distribution of PD participants in this cohort resulted in 40.7% classified as cognitively intact, 12.3% as left frontosubcortical, 11.1% as right frontosubcortical, 8.6% as global, 8.6% as left posterior, 7.4% as right posterior, 6.2% as left hippocampal, as 4.9% right hippocampal (Table 3).

## 4. Discussion

MRI measures, age, and education predict cognitive decline in PD [22]. Vasconcellos et al. (2017) investigated the relationship between MRI visual rating scales and the neuropsychological profile in patients with PD-MCI [23]. The study included 79 PD patients and 92 controls, assessing Global Cortical Atrophy (GCA), Medial Temporal Atrophy (MTA), and the Fazekas scale for white matter lesions. While no significant differences were found in the frequency of MRI visual scale abnormalities between amnestic and non-amnestic subtypes, inverse correlations were identified between GCA scores and cognitive domains such as memory, attention, language, and executive function [23]. The authors suggest that MRI visual rating scales may be more useful in cases of more advanced MCI or PDD. Our study, using more visual scales and extended neuropsychological battery, demonstrates the relevance of mesial temporal, frontocortical, and cingulate atrophy—assessed via visual rating scales—and their associations with executive, visuospatial, and global cognitive performance in early-stage PD. These results align with previous cross-sectional studies using volumetric MRI, such as Brandão et al. [24], which also identified atrophy in frontoinsular, mesial temporal, and cingulate regions as strongly linked to cognitive impairment in PD. Moreover, our findings demonstrate the modulatory effects of demographic variables on cognition: older age was associated with poorer performance in planning and interference control, while higher education attenuated deficits in verbal fluency and attention. These observations are consistent with prior evidence supporting the protective role of cognitive reserve in PD [25,26] and emphasize the relevance of demographic context in risk stratification and clinical interpretation. Moreover, our findings support the presence of cognitive impairment within the first three years of PD diagnosis in some cases and highlight the utility of MoCA and PD-CRS as sensitive screening tools in early stages.

Importantly, this work demonstrates that distinct cognitive–anatomical subtypes can be identified in early-stage PD through the integration of structured neuropsychological assessments and regional atrophy as quantified by visual MRI scales. Rather than representing a single clinical phenotype, PD emerges as a spectrum of syndromes with spatially divergent cortical and subcortical degeneration. The eight anatomo-cognitive subtypes derived through our constrained clustering approach exhibited anatomical plausibility and internal consistency, with post hoc cross-validated AUC values ranging from 0.68 to 0.95. While the use of theoretical centroids introduces prior knowledge into the clustering process, our sensitivity analyses suggest that the subtype structure is robust and not unduly dependent on seed selection. This anatomically grounded approach may balance interpretability and flexibility in characterizing cognitive heterogeneity in early PD.

These patterns reinforce the notion that cognitive impairment in PD is multifactorial and regionally distributed. In particular, our results confirmed strong associations between medial temporal atrophy and episodic memory deficits, orbitofrontal degeneration and executive dysfunction, and posterior cortical involvement and visuospatial impairment. Such findings echo previous studies that delineated frontostriatal and posterior cortical cognitive syndromes in PD [7,8]. Notably, our use of brief, validated visual rating scales—originally developed for Alzheimer’s disease and increasingly used in PD—proved sensitive to clinically relevant cognitive variability [20,21]. In line with our results, a recent study by Fornari et al. [27] also found that medial temporal atrophy measured by visual scales effectively differentiated cognitively impaired PD patients from cognitively unimpaired individuals, and that occipital atrophy further distinguished them from healthy controls. These findings suggest that this classification framework could inform future studies on early patient stratification and prognostic assessment.

The observed regional atrophy patterns involve structures implicated not only in executive and mnemonic processes but also in self-related cognitive functions, such as agency and identity. Networks such as the salience and default mode networks—encompassing the anterior cingulate, orbitofrontal cortex, and insula—are increasingly recognized for their roles in modulating self-awareness and dynamic cognitive control [28,29]. The distribution of participants across subtypes further highlights the multidimensional heterogeneity of early PD, with the global subtype showing broader atrophic involvement and more pronounced cognitive deficits. While these findings underscore the internal coherence of the proposed classification, their prognostic and translational value requires prospective validation.

Finally, our subtype-based framework builds upon recent studies that emphasize the neurobiological heterogeneity of PD. For instance, Iguanzo et al. [30] applied unsupervised clustering to cortical thickness data in early PD and identified eight structural subtypes with distinct cognitive signatures and severity levels. Our anatomically guided, hybrid approach—combining theory-based correspondence matrices with k-means refinement—produced similar subgroup structures, including clearly defined posterior and frontosubcortical clusters. The convergence between our results and those of Iguanzo and colleagues reinforces the plausibility of neuroanatomically informed classification models in early PD.

Importantly, our data suggest that early cognitive–anatomical subtypes may provide a foundation for future studies exploring prognostic implications. The posterior and hippocampal phenotypes, though less prevalent in our sample, were associated with more severe cognitive impairment and may represent trajectories with increased risk of progression to dementia. These findings align with results from longitudinal cohorts such as PPMI and ICICLE-PD, where early deficits in visuospatial and semantic domains were predictive of accelerated decline [31]. Similarly, our hippocampal subtypes share key features with the “amnestic” profiles described by Labrador-Espinosa et al. in PDD, who reported a higher frequency of GBA mutations and visual hallucinations in patients with prominent visuospatial deficits [32].

The regional atrophy patterns identified in this study provide structural support for existing neuropathological staging models of PD. In particular, our findings are consistent with the Braak hypothesis, which posits a progression of Lewy pathology from brainstem to limbic and, eventually, neocortical regions [5]. The emergence of distinct frontosubcortical and posterior cognitive syndromes in early-stage PD may reflect this transition toward neocortical involvement. Moreover, the relative preservation of mesial temporal structures in the majority of patients aligns with evidence suggesting that limbic degeneration in PD occurs later and less consistently than in Alzheimer’s disease [33]. Nevertheless, the detection of isolated hippocampal atrophy in a subset of individuals points to the potential presence of co-pathologies such as amyloid-β or tau deposition, which may be active even in prodromal or early clinical stages. This is corroborated by recent biomarker research showing Alzheimer-like CSF profiles in a subset of PD patients with early memory impairment [34].

While our study provides cross-sectional insights into cognitive and neuroimaging profiles in PD, we acknowledge the importance of complementing static classifications with dynamic and longitudinal perspectives. Neurodegenerative progression in PD is inherently nonlinear and shaped by brain-based factors, such as compensatory plasticity and self-network reconfiguration, as well as context-dependent influences like cognitive reserve and technological mediation of cognition [35,36]. Emerging frameworks suggest that trajectories of self-related decline may better capture the heterogeneity of PD and enhance prognostic accuracy compared to static diagnostic labels [37,38]. Moreover, dynamic reorganization within the cortical midline structures and intrinsic connectivity networks—including the default mode and salience networks—has been implicated in self-referential processing and may hold relevance for early stratification in PD [28,29,39]. Future research integrating longitudinal self-modeling approaches could thus substantially refine the taxonomy proposed here, allowing for a more nuanced understanding of patient trajectories.

## 5. Conclusions

This study identifies cognitive–anatomical phenotypes in early-stage PD by integrating neuropsychological profiles with region-specific atrophy assessed via validated visual MRI scales. These subtypes, derived through a constrained clustering approach, reflect clinically plausible and internally consistent groupings.

While exploratory, this framework highlights the potential of cognitive screening and visual MRI markers for characterizing heterogeneity in early PD. Future validation in larger, longitudinal cohorts, with biomarker and functional outcome integration, will be essential to establish its prognostic and clinical utility.

## Figures and Tables

**Table 1:** Demographic variables, handedness, and education in the HUCA cohort.

Variable	Cases (n = 81)	Controls (n = 20)	Total	Statistic	df	*p*	Effect Size—Cohen’s d-(95%CI)
Mean age (SD)	66.46 (10.86)	62.05 (11.02)	65.58 (10.97)	U = 1018.5		0.075	0.404
Men/Women	45/36	8/12	53/48	χ^2^ = 0.99504	1	0.3185	
Laterality (right/left/ambidextrous)	78/2/1	18/2/0	96/4/1	χ^2^ = 2.6107	2	0.2711	
Education							
Primary	14	7	21				
Secondary	34	11	45				
High school/Vocational training	11	1	12				
Pre-University course	8	0	8				
University degree	5	1	6				
Doctorate	9	0	9				
MoCA total (SD)	22.06 (4.24)	27.15 (1.69)	23.07 (4.37)	U = 195.5		<0.001	−1.30 (−1.83–0.78)
PD-CRS subcortical (SD)	55.74 (13.90)	87.25 (7.12)	61.98 (18.01)	t = −14.17	59.30	<0.001	−2.43 (−3.03–1.83)
PD-CRS cortical	26.96 (3.19)	29.90 (0.31)	27.54 (3.09)	U = 192		<0.001	−1.02 (−1.53, −0.50)
PD-CRS total (SD)	82.70 (15.78)	117.15 (7.20)	89.52 (19.98)	t = −14.56	69.5	<0.001	−2.36 (−2.96, −1.77)

**Table 2:** Significant correlations between neuropsychological variables †.

Variable	r_1_	p_1_	r_2_	p_2_	r_3_	p_3_	N
Tower of Hanoi (moves)	−0.433 **	<0.001	−0.420 **	0.001	−0.444 **	<0.001	63
Tower of Hanoi (time)	−0.658 **	<0.001	−0.520 **	<0.001	−0.663 **	<0.001	63
Judgment of Line Orientation (JLO)	0.754 **	<0.001	0.504 **	<0.001	0.756 **	<0.001	80
Cancellation	0.120	0.287	0.154	0.171	0.131	0.244	81
Phonemic fluency	0.789 **	<0.001	0.458 **	<0.001	0.781 **	<0.001	81
Semantic fluency	0.664 **	<0.001	0.376 **	0.001	0.656 **	<0.001	81
Action fluency	0.750 **	<0.001	0.395 **	<0.001	0.737 **	<0.001	81
Action naming	0.641 **	<0.001	0.703 **	<0.001	0.684 **	<0.001	81
Visual memory delayed recall	0.586 **	<0.001	0.453 **	<0.001	0.597 **	<0.001	80
Symbol Digit Modalities Test (SDMT)	0.856 **	<0.001	0.595 **	<0.001	0.862 **	<0.001	81
Delayed recall hits	0.658 **	<0.001	0.522 **	<0.001	0.673 **	<0.001	81
MoCA total score	0.760 **	<0.001	0.533 **	<0.001	0.768 **	<0.001	101
True recognition hits	0.408 **	<0.001	0.158	0.160	0.392 **	<0.001	81

† r_1_/p_1_, r_2_/p_2_, and r_3_/p_3_ refer, respectively, to correlations with the PD-CRS subcortical, cortical, and total scores. ** *p* < 0.001.

**Table 3:** Distribution of subtypes in our cohort.

Subtype	n	% of Total
Cognitively intact	33	40.7%
Left frontosubcortical	10	12.3%
Right frontosubcortical	9	11.1%
Left posterior	7	8.6%
Right posterior	6	7.4%
Left hippocampal	5	6.2%
Right hippocampal	4	4.9%
Global	7	8.6%
Total	81	100%

## Data Availability

The raw data supporting the conclusions of this article will be made available by the authors on request.

## Supplementary Materials

The following supporting information can be downloaded at: https://www.mdpi.com/article/10.3390/brainsci15070751/s1, Supplementary Figure S1: Heatmap of median visual MRI ratings by cluster; Supplementary Figure S2: Distribution of silhouette scores; Supplementary Table S1. Cognitive–Visual MRI Correlations (Right Hemisphere); Supplemental Table S2. Cognitive–Visual MRI Correlations (Left Hemisphere); Supplementary Table S3. MANOVA Cases vs. Controls: Regional Atrophy Differences; Supplementary Table S4. MANOVA by Cognitive Impairment Group (PD-CRS); Supplementary Table S5. Correlations vMRI ↔ PD-CRS; Supplementary Table S6. Most informative tests and subtests for each anatomo-cognitive subtype; Supplementary Table S7: Median visual MRI ratings by cluster; Supplementary Table S8. Standardized neuropsychological z-scores by cluster.
