# Supplementary material for: Subtyping Early Parkinson’s Disease by Mapping Cognitive Profiles to Brain Atrophy with Visual MRI Ratings
Source: Brain Sci. 2025 Jul 15;15(7):751. doi: 10.3390/brainsci15070751 (PMC12294059; doi:10.3390/brainsci15070751)
Supplement: Supplementary file 1 [file brainsci-15-00751-s001.zip › brainsci-3682500-supplementary.pdf]

Supplemental file

Supplementary tables

Supplementary Table S1. Cognitive–Visual MRI Correlations (Right Hemisphere)

**Cognitive Variable**	**Medial Temporal**	**Anterior Temporal**	**Fronto-Insular**	**Orbitofrontal**	**Anterior Cingulate**	**Posterior Cortex**
Stroop Interference	0.072	0.050	–0.046	–0.016	–0.055	–0.103
Tower of Hanoi (Moves)	0.162	0.172	0.156	0.257 *	0.278 *	–0.024
Tower of Hanoi (Time)	0.158	0.417 **	0.204	0.281 *	0.346 **	–0.034
Judgment of Line Orientation	–0.451 **	–0.188	–0.291 **	–0.359 **	–0.459 **	–0.307 **
Cancellation	–0.196	–0.066	–0.004	–0.204	–0.241 *	–0.440 **
Phonemic Fluency (“F”)	–0.230 *	–0.297 **	–0.404 **	–0.358 **	–0.383 **	–0.197
Semantic Fluency	–0.180	–0.298 **	–0.288 **	–0.233 *	–0.243 *	–0.157
Action Fluency	–0.253 *	–0.369 **	–0.293 **	–0.273 *	–0.327 **	–0.215
Action Naming	–0.444 **	–0.240 *	–0.293 **	–0.340 **	–0.399 **	–0.265 *
Delayed Visual Memory	–0.301 **	–0.411 **	–0.320 **	–0.187	–0.238 *	–0.133
SDMT	–0.389 **	–0.317 **	–0.417 **	–0.435 **	–0.466 **	–0.292 **
HVLT-R Recognition Hits	–0.464 **	–0.348 **	–0.232 *	–0.206	–0.369 **	–0.216
HVLT-R Recognition (Yes/No)	–0.348 **	–0.314 **	–0.094	–0.079	–0.179	–0.220 *
MoCA Total Score	–0.436 **	–0.308 **	–0.317 **	–0.365 **	–0.430 **	–0.234 *

* $p < .05$; ** $p < .01$

Supplementary Table S2. Cognitive–Visual MRI Correlations (Left Hemisphere)

Cognitive Variable	Medial Temporal	Anterior Temporal	Fronto-Insular	Orbitofrontal	Anterior Cingulate	Posterior Cortex
Stroop Interference	0.013	–0.005	–0.056	–0.025	–0.136	0.024
Tower of Hanoi (Moves)	0.393 **	0.137	0.081	0.177	0.099	0.059
Tower of Hanoi (Time)	0.367 **	0.231	0.152	0.240	0.169	–0.010
Judgment of Line Orientation	–0.457 **	–0.124	–0.300 **	–0.305 **	–0.413 **	–0.380 **
Cancellation	–0.196	–0.063	0.054	–0.126	–0.314 **	–0.467 **
Phonemic Fluency (“F”)	–0.382 **	–0.192	–0.282 *	–0.318 **	–0.238 *	–0.195
Semantic Fluency	–0.326 **	–0.170	–0.169	–0.228 *	–0.165	–0.251 *
Action Fluency	–0.310 **	–0.232 *	–0.260 *	–0.248 *	–0.210	–0.270 *
Action Naming	–0.351 **	–0.164	–0.268 *	–0.265 *	–0.261 *	–0.267 *
Delayed Visual Memory	–0.320 **	–0.182	–0.157	–0.247 *	–0.179	–0.123
SDMT	–0.417 **	–0.235 *	–0.364 **	–0.377 **	–0.420 **	–0.314 **
HVLT-R Recognition Hits	–0.488 **	–0.313 **	–0.300 **	–0.349 **	–0.334 **	–0.237 *
HVLT-R Recognition (Yes/No)	–0.247 *	–0.335 **	–0.284 *	–0.114	–0.157	–0.233 *
MoCA Total Score	–0.379 **	–0.214 *	–0.321 **	–0.352 **	–0.352 **	–0.307 **

* $p < .05$; ** $p < .01$

Supplementary Table S3. MANOVA Cases vs. Controls: Regional Atrophy Differences (Visual Rating)

Region (Visual Scale)	F	p	Partial η^2
Medial Temporal (R)	9.523	0.003	0.088
Anterior Temporal (R)	11.226	0.001	0.102
Fronto-Insular (R)	9.931	0.002	0.091
Orbitofrontal (R)	13.154	< 0.001	0.117
Anterior Cingulate (R)	13.481	< 0.001	0.120
Posterior Cortex (R)	5.591	0.020	0.053
Medial Temporal (L)	13.693	< 0.001	0.122
Anterior Temporal (L)	5.200	0.025	0.050
Fronto-Insular (L)	10.453	0.002	0.096
Orbitofrontal (L)	15.348	< 0.001	0.134
Anterior Cingulate (L)	7.937	0.006	0.074
Posterior Cortex (L)	4.734	0.032	0.046

Supplementary Table S4. MANOVA by Cognitive Impairment Group (PD-CRS)

Region (Visual Scale)	F	p	Partial η^2	Trend
Anterior Cingulate (R)	8.811	< 0.001	0.154	↑ atrophy
Fronto-Insular (R)	5.784	0.004	0.107	↑
Medial Temporal (R)	5.103	0.008	0.095	↑
Anterior Temporal (R)	4.946	0.009	0.093	↑
Orbitofrontal (R)	4.297	0.016	0.081	↑
Medial Temporal (L)	4.841	0.010	0.091	↑
Fronto-Insular (L)	3.190	0.046	0.062	↑
Orbitofrontal (L)	3.198	0.045	0.062	↑
(Posterior cortex and anterior cingulate left not significant)				

Supplementary Table S5. Correlations vMRI ⇔ PD-CRS”

5 A. Right hemisphere

Region	*Subcortical*	*Cortical*	*Total*
Medial Temporal	–0.406 **	–0.357 **	–0.421 **
Anterior Temporal	–0.362 **	–0.173	–0.353 **
Fronto-Insular	–0.421 **	–0.216 *	–0.413 **
Orbito-Frontal	–0.377 **	–0.229 *	–0.375 **
Anterior Cingulate	–0.468 **	–0.335 **	–0.474 **
Posterior Cortex	–0.253 *	–0.255 *	–0.268 **

5 B. Left hemisphere

Region	*Subcortical*	*Cortical*	*Total*
Medial Temporal	–0.446 **	–0.330 **	–0.453 **
Anterior Temporal	–0.267 **	–0.018	–0.243 *
Fronto-Insular	–0.368 **	–0.122	–0.351 **
Orbito-Frontal	–0.358 **	–0.177	–0.350 **
Anterior Cingulate	–0.313 **	–0.178	–0.310 **
Posterior Cortex	–0.240 *	–0.213 *	–0.249 *

Supplementary Table S6. Most informative tests and subtests for each anatomo-cognitive subtype

Subtype	MRI Atrophy (Visual Scales)	Predominant Functions	Tests/Subtests
Cognitively intact	No atrophy (all scales scored 0–1)	Global function	Full neuropsychological battery
Left frontosubcortical	Left frontoinsular · Left orbitofrontal · Left anterior cingulate	Sustained attention · Verbal working memory · Fluency and inhibition control	PD-CRS Sustained Attention · PD-CRS Verbal Working Memory · PD-CRS Alternating Verbal Fluency · PD-CRS Action Fluency · MoCA Digit Span Backward · MoCA Serial 7's · Phonemic Fluency · Stroop Interference · Action Naming · Tower of Hanoi Moves and Time · Cancellation Task (letters/numbers) · Lexical Decision/Semantic Priming
Right frontosubcortical	Right frontoinsular · Right orbitofrontal · Right anterior cingulate	Visuospatial attention and working memory · Visuospatial planning	PD-CRS Clock Drawing (spontaneous) · PD-CRS Clock Copy · PD-CRS Sustained Attention · MoCA Cube Copy · MoCA Clock Drawing · MoCA Digit Span Forward · JLO · Tower of Hanoi · Visual Cancellation Task
Left posterior	Left medial temporal · Left anterior temporal · Left posterior cortex	Lexical-semantic · Calculation · Verbal reasoning	PD-CRS Confrontation Naming · PD-CRS Alternating Verbal Fluency · PD-CRS Delayed Verbal Memory · MoCA Animal Naming · MoCA Abstraction · MoCA Serial 7's · MoCA Delayed Recall · Semantic Fluency (animals, supermarket) · HVLT-R Delayed Recall · HVLT-R Recognition · Lexical Decision / Semantic Priming
Right posterior	Right medial temporal · Right anterior temporal · Right posterior cortex	Visuoperception · Visual attention	PD-CRS Clock Copy · PD-CRS Clock Drawing (spontaneous) · MoCA Cube Copy · MoCA Clock Drawing · JLO · Visual Cancellation Task · Stroop Color Naming
Left hippocampal	Left mesial temporal atrophy (±parahippocampal extension)	Verbal episodic memory	PD-CRS Delayed Verbal Memory · PD-CRS Immediate Verbal Memory · HVLT-R Immediate Recall · HVLT-R Delayed Recall · HVLT-R Recognition · MoCA Delayed Recall
Right hippocampal	Right mesial temporal atrophy (±parahippocampal extension)	Visuospatial episodic memory	JLO · MoCA Cube Copy · MoCA Clock Drawing · PD-CRS Clock Copy (proxy)
Global	Multiregional atrophy (≥4 regions affected)	Deficits in ≥4 cognitive domains	Full neuropsychological battery

Supplementary Table S7. Median Visual MRI Ratings by Cluster

Median scores for 12 visual MRI rating scales across the eight cognitive–anatomical subtypes. Higher scores indicate greater regional atrophy.

	Medial Temporal	Posterior	Orbitofrontal	Anterior Cingulate	Frontoinsular	Frontal	Parietal	Occipital	Temporal Pole	Hippocampal	Amygdala	Insula
Cognitively intact	1.85	1.40	1.95	2.57	1.34	1.34	2.61	2.04	1.17	1.88	1.18	1.17
Left fronto-subcortical	1.67	0.16	0.29	1.11	0.79	1.72	0.86	0.51	2.53	1.34	1.55	0.50
Right fronto-subcortical	1.12	1.58	0.69	1.76	1.08	1.30	1.08	2.80	1.49	0.76	2.08	0.65
Left posterior	1.65	0.13	0.57	1.64	2.02	1.62	1.42	1.29	0.47	1.00	1.18	2.24
Right posterior	1.74	0.27	1.73	1.23	1.03	1.93	2.22	2.15	0.91	1.28	1.73	2.18
Left hippocampal	1.16	1.37	0.73	0.66	2.07	2.45	1.45	2.20	1.75	1.05	1.75	2.58
Right hippocampal	1.47	2.60	0.00	2.08	1.56	1.29	1.56	0.11	1.35	1.75	2.53	1.14
Global	0.93	1.15	2.14	1.73	1.13	1.86	1.57	2.18	1.01	1.27	1.23	0.48

Supplementary Table S8. Standardized Neuropsychological Z-scores by Cluster

Mean z-scores for 10 key cognitive domains in each cluster centroid. Negative values reflect lower performance relative to normative data.

	Phonemic Fluency	Semantic Fluency	Action Naming	Tower of Hanoi	JLO	SDMT	Stroop Interference	Digit Span	MoCA Total	HVLT-R Delayed Recall
Cognitively intact	0.30	0.26	0.01	-0.23	-1.42	-0.42	-0.34	-0.80	-0.16	0.40
Left fronto-subcortical	1.89	0.17	0.26	-0.07	-1.92	-0.03	0.06	2.00	-0.19	0.30
Right fronto-subcortical	-0.03	-1.17	1.14	0.75	0.79	-0.91	1.40	-1.40	0.59	2.00
Left posterior	-0.99	-0.57	0.10	-0.50	-1.55	0.07	-1.06	0.47	-0.92	1.55
Right posterior	-0.78	-0.32	0.81	-1.23	0.23	1.31	-1.61	0.18	0.26	0.78
Left hippocampal	-1.24	-1.32	0.52	0.30	0.25	0.35	-0.68	0.23	0.29	-0.71
Right hippocampal	1.87	0.47	-1.19	0.66	-0.97	0.79	1.16	-0.82	0.96	0.41
Global	0.82	1.90	-0.25	-0.75	-0.89	-0.82	-0.08	0.34		

Supplementary Figures

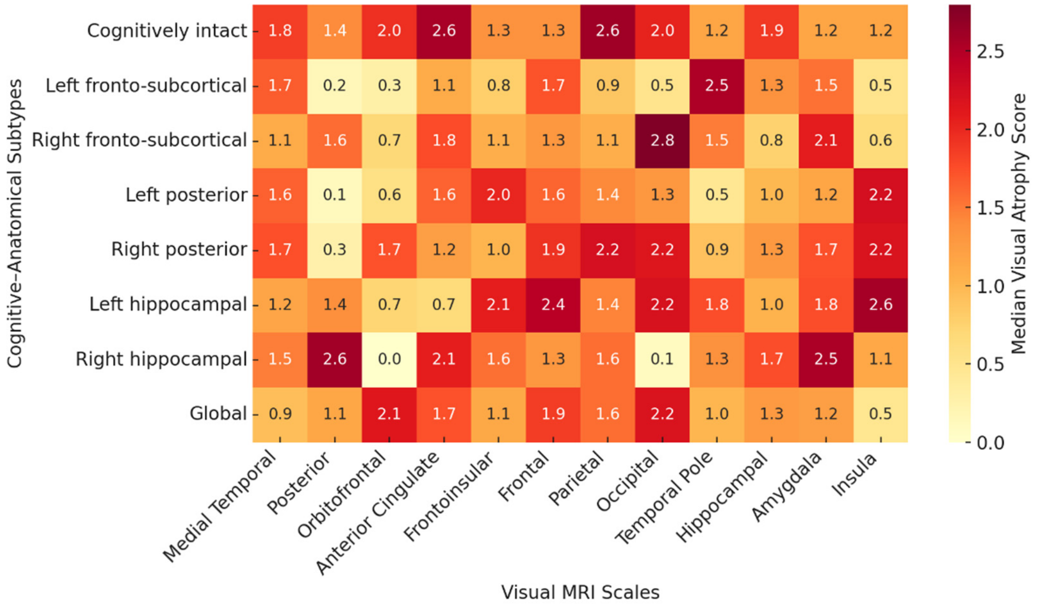

Supplementary Figure S1. Heatmap of Median Visual MRI Ratings by Cluster
A heatmap depicting the median atrophy scores across 12 visual MRI scales for the eight cognitive–anatomical subtypes. Darker colors indicate greater atrophy.

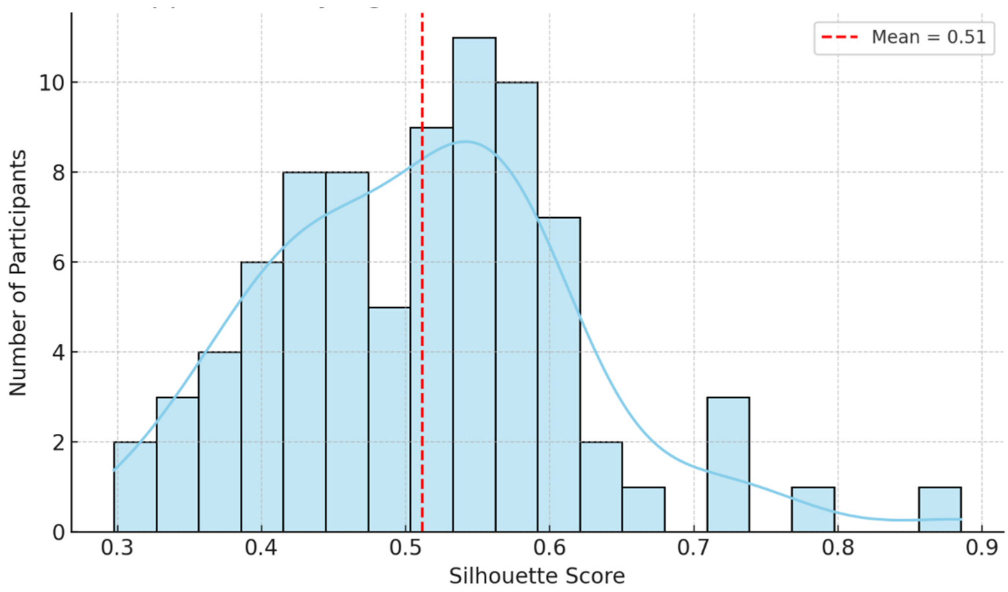

Supplementary Figure S2. Distribution of Silhouette Scores. Histogram of silhouette scores for all participants in the PD cohort showing cluster assignment quality. The dashed red line indicates the mean silhouette score across clusters.
